# Extrinsic Calibration of a Laser Galvanometric Setup and a Range Camera

**DOI:** 10.3390/s18051478

**Published:** 2018-05-08

**Authors:** Seppe Sels, Boris Bogaerts, Steve Vanlanduit, Rudi Penne

**Affiliations:** 1 Departement Electromechanics, Faculty of Applied Engineering, University of Antwerp, 2020 Antwerpen, Belgium; boris.bogaerts@uantwerpen.be (B.B.); steve.vanlanduit@uantwerpen.be (S.V.); rudi.penne@uantwerpen.be (R.P.); 2 Departement of Mathematics, University of Antwerp, 2020 Antwerpen, Belgium

**Keywords:** galvanometer, range camera, extrinsic calibration, Non-Perspective-n-Point Problem, scanning laser Doppler vibrometer

## Abstract

Currently, galvanometric scanning systems (like the one used in a scanning laser Doppler vibrometer) rely on a planar calibration procedure between a two-dimensional (2D) camera and the laser galvanometric scanning system to automatically aim a laser beam at a particular point on an object. In the case of nonplanar or moving objects, this calibration is not sufficiently accurate anymore. In this work, a three-dimensional (3D) calibration procedure that uses a 3D range sensor is proposed. The 3D calibration is valid for all types of objects and retains its accuracy when objects are moved between subsequent measurement campaigns. The proposed 3D calibration uses a Non-Perspective-n-Point (NPnP) problem solution. The 3D range sensor is used to calculate the position of the object under test relative to the laser galvanometric system. With this extrinsic calibration, the laser galvanometric scanning system can automatically aim a laser beam to this object. In experiments, the mean accuracy of aiming the laser beam on an object is below 10 mm for 95% of the measurements. This achieved accuracy is mainly determined by the accuracy and resolution of the 3D range sensor. The new calibration method is significantly better than the original 2D calibration method, which in our setup achieves errors below 68 mm for 95% of the measurements.

## 1. Introduction and Related Work

Laser galvanometric systems (abbreviated to laser galvanometers) are systems commonly used for laser projection systems, optical measurement devices, and projection based systems. In this paper, a scanning laser Doppler vibrometer (Polytec PSV 300) laser galvanometer system is used [1,2]. A laser Doppler vibrometer (LDV) is an optical measurement device designed to measure vibrations of an object. By using a galvanometer, the instrument is able to move the laser spot to different positions on the object.

**Figure 1:**
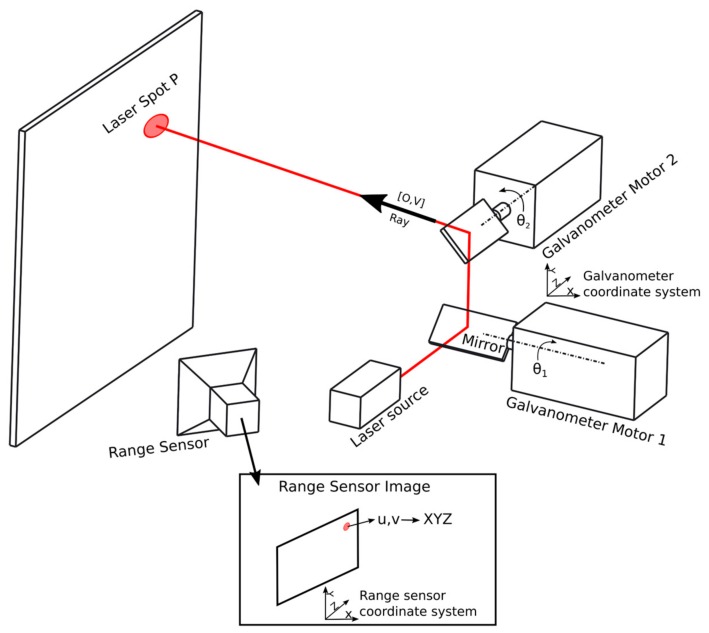
Schematic overview of the galvanometric mirror system and range sensor.

Laser galvanometers generally consist of two movable mirrors (Figure 1). The angle of a mirror can be altered by a galvanometer motor. These moving mirrors are used to deflect a laser beam to a certain position. Laser galvanometers can be used in combination with camera systems to provide feedback about the laser spot position. When the laser galvanometer system and the camera system are extrinsically calibrated, the laser galvanometer can aim the laser spot to a position predefined in the camera coordinate system. Extrinsic calibration calculates the position (rotation and translation) between the camera and laser galvanometer in a reference coordinate system. This calibration assumes that the intrinsic parameters of both the laser galvanometer and camera are known.

Current laser galvanometers use a two-dimensional (2D) camera and a calibration procedure which is valid only for planar objects to automatically position a laser dot towards a particular point on an object under test [3]. This method is limited because it assumes that measurements are made in a planar region. This assumption does not hold for nonplanar or moving objects [4,5].

In our setup, we replace the traditionally used 2D camera with a three-dimensional (3D) range sensor. The 3D range sensor can then provide the 3D coordinate of a laser spot location on an object. With a 3D extrinsic calibration between the 3D range sensor and the laser galvanometer, the laser galvanometer can aim the laser at arbitrary 3D target locations on the object. The 3D extrinsic calibration will retain its validity, even when target objects are moved. The fast and accurate extrinsic calibration setup that is proposed in this article is especially useful in mobile setups where the 3D range sensor and laser galvanometer are not mounted in a fixed setup. This allows us to position both the galvanometer system and the 3D range sensor at their optimal position (to achieve a maximum accuracy). Our method also enables the use of different and/or multiple range sensors.

Other calibration systems exist and are described in literature. These systems mostly use the galvanometer as a 3D range sensor instead of in combination with a 3D range sensor [6,7]. Moreover, when these systems are combined with camera systems, the camera remains fixed in the setup. Our methodology allows the range sensor to move between setups with only a minimal extrinsic calibration procedure. Systems like lidar directly measure the length of a laser ray to an object. This reduces the calibration to a standard calibration between range sensors [7,8] or to a calibration between a range sensor and 2D camera [9,10].

A 3D range sensor like a Time-of-Flight camera can be used to detect the 3D pose of an object under test. Although this is not done in this work, this pose can be used to automatically aim the laser galvanometer to a predefined point on the object. Pose estimation techniques with 3D range sensors exist and some are available, such as PCL-C++ libraries [11,12,13]. These techniques can also be used to track moving objects. For increased accuracy, other sensors such as RGB cameras, MARG-sensors, and IMU-sensors can be used in combination with a range sensors [14,15].

In this work, a 3D extrinsic calibration method between a laser galvanometric setup and a 3D range sensor is proposed. In Section 2, the main algorithm is explained together with the calibration methodology. Also, the procedure to test the proposed calibration methodology is explained. In Section 3 the experimental setup and the experimental results are presented. We also include an extensive stability analysis of the algorithm (which allows us to derive a set of specific recommendations for obtaining a good calibration). In addition, it is shown that our proposed 3D calibration method significantly outperforms the traditionally used 2D calibration method of [3].

## 2. Algorithm and Methodology

In this section, an overview of the general setup and methodology is given. Next, the main algorithm used to solve this problem is briefly explained. Following that, the complete methodology that uses this algorithm is explained. Finally, the validation procedure (used in our experiments) of the methodology is explained.

### 2.1. Schematic Overview of the General Setup and Problem

Figure 1 gives a general overview of the laser galvanometric system and a range sensor. The direction of the outgoing laser ray of the laser galvanometer is controlled by two galvanometer motors driving two mirrors. In this work, we describe the laser ray by a start point (*O_i_*) and its direction (*V_i_*). The mapping of the galvanometer motor angles (θ) to the line-coordinates of the corresponding ray can be provided by the manufacturer, or by methods like the ones proposed by Manakov et al. [5] or Cui et al. [6] (in this work, we use the work of Cui et al.). These laser rays are described in a coordinate system specific for the laser galvanometer. The laser spot (*P_i_)* is detected by a range sensor in the range sensor coordinate system. The goal in this work is to calculate the transformation between these two coordinate systems. Calculating this transformation is called the extrinsic calibration between the range sensor and laser galvanometer. This calibration is abbreviated to ‘3D calibration’ in this work.

When the laser ray is aimed towards an object, the range sensor can see a laser spot (*P_i_*). In our work, this laser spot is detected in [*u*,*v*]-coordinates and then converted to *XYZ*-coordinates in the range sensor coordinate system using the range sensor capabilities. For the methodology, it is not important how these *XYZ*-coordinates of the laser spot are obtained. How this is done in our experimental setup is explained in Section 3.

We define a “Sample Point” as a tuple containing the following data:

1. *O_i_*, *V_i_* (start point and direction) of the laser ray in the laser galvanometer coordinate system.
2. *XYZ_i_*-coordinate of the detected laser spot *P_i_* of the corresponding ray in the range sensor coordinate system.

This definition of a sample point will be used in the remainder of this paper.

### 2.2. Overview Methodology

**Figure 2:**
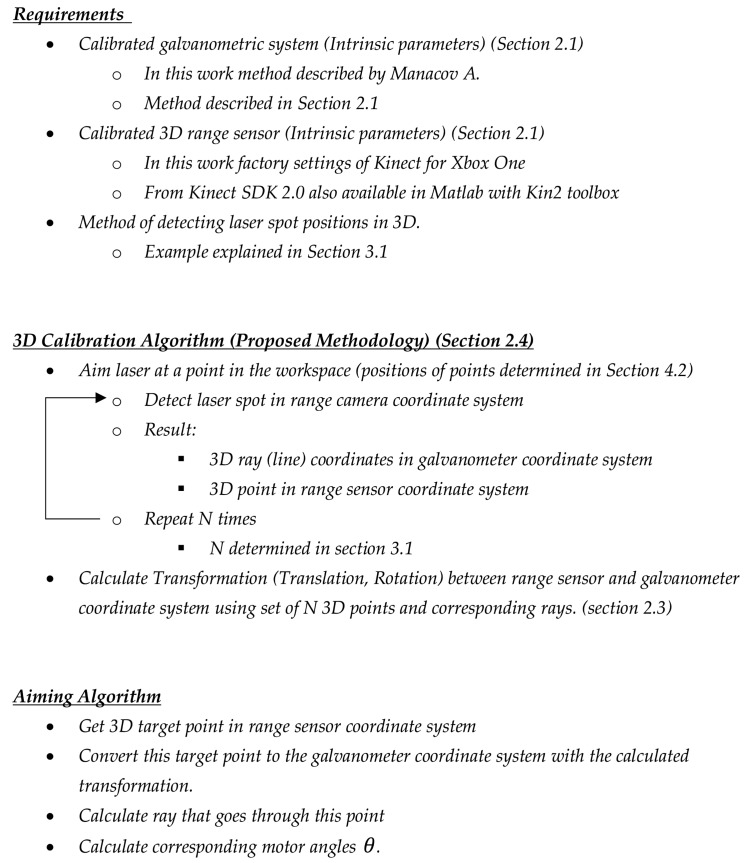
Overview of the complete proposed process used in the paper from preconditions [16,17,18] and three-dimensional (3D) calibration to aiming towards a selected point [19].

In Figure 2, a schematic overview of the steps and preconditions of the complete process in the paper is given.

### 2.3. Non-Perspective-n-Point Problem Solution

**Figure 3:**
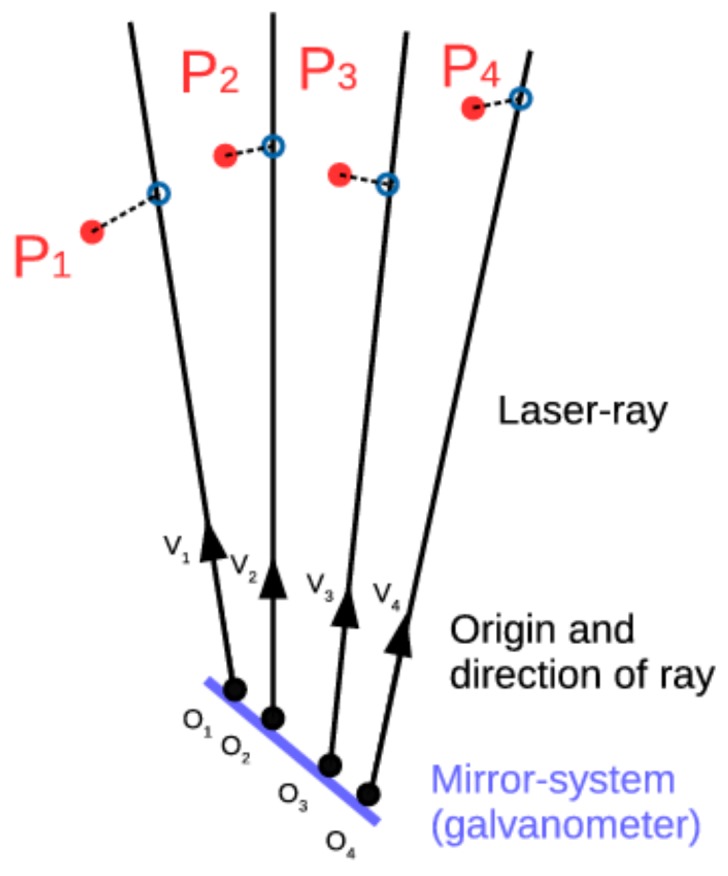
Representation of the non-Perspective-n-Point problem with a laser galvanometer. The laser rays (*O_i_*, *V_i_*) are described in the laser galvanometer coordinate system while laser dots (*P_i_*) are described in the range sensor coordinate system.

The goal of the 3D calibration procedure is to calculate the coordinate transformation (rotation and translation) between the laser galvanometer coordinate system and the 3D range sensor coordinate system. From the laser galvanometer, only the rays (defined by a start point (*O_i_*) and direction (*V_i_*)) are known in the laser galvanometer coordinate system. The laser spot positions (*P_j_*) on the object are known in the 3D coordinate system of the 3D range sensor. Due to the offset between the two mirrors in the mirror-system of the laser galvanometer, the outgoing rays do not intersect in one point (see Figure 3). Therefore, a standard solution of the Perspective-n-Point (PnP) problem, as commonly used in camera-calibration, cannot be used to calculate the coordinate transformation [20,21]. In this work, the Non-Perspective-n-Point (NPnP) problem solution of Fusiello et al. [22] is used to overcome this problem. This solution searches for the coordinate-transformation that minimizes the distance between the rays in the laser galvanometer-coordinate system and the points in the 3D range sensor coordinate system. This Non-Perspective-n-Point problem is a generalization of the traditional Perspective-n-Point problem where outgoing rays are not constrained to intersect in one specific point (the focal point of a camera).

As is the case in the standard PnP-problem, the distance (*d_i_*) of Equation (1) is minimized. In this work, the procrustean solution of Fusiello et al. [22] is used. *T* and *R* are the translation and rotation matrix, respectively, from the 3D range sensor coordinate system to the laser galvanometer coordinate system. *E* is the cost-function (given in Equation (2)) that is minimized and *δ_i_* defines the distance of a 3D point *P_i_* along with a corresponding line (*O_i_*; *V_i_*). Note that in the original work of Fusiello et al. [22], a scaling parameter is calculated, but this is not needed in this work. We use the iterative solution presented by Fusiello et al. [22] to solve the nonlinear optimization problem. Fusiello et al. [22] showed that this solution systematically converges for arbitrary starting values *δ_i_* (while this is not the case for their direct solution). We used *δ_i_* = 1 as a starting value.

We define the following distance function between a laser ray and a laser spot:

(1) $$d_{i}\left(T,R,i\right)=\left(\delta_{i}V_{i}+O_{j}\right)-\left(RP_{i}+T\right)$$

which allows us to define the cost function *E*:

(2) $$\underset{\alpha }{E=\mathrm{argmin}}\;{\left||d_{i}(T,R,\delta_{i})|\right|}_{2}$$

(3) $$\alpha =\left[\begin{array}{ccc} vec\left(R\right) & T & \delta_{i} \end{array}\right]$$

In order to solve the NPnP problem, the following requirements must be met:

1. The coordinates (*O_i_*; *V_i_*) of the rays of the galvanometric system are known. These coordinates can be obtained by using one of the methods described in [16,17,23]. In this work, the parameters of the galvanometric system are obtained by the method described in [16].
2. The used a 3D sensor is able to detect the laser spot of the galvanometric system and can calculate the position of this spot in a 3D coordinate system. The detection used in this work is explained in Section 3 (Experimental Setup).

### 2.4. Methodology of the 3D Extrinsic Calibration

**Figure 4:**
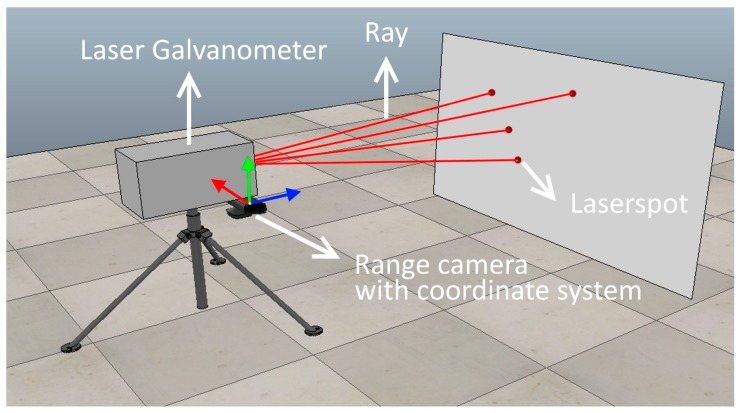
Example of calibration setup. The laser rays are described in the laser galvanometer coordinate system. The laser spots are detected and described in the 3D range camera coordinate system.

Step 1: In the first step, the laser galvanometer aims the laser spot at N arbitrary points on a plate (see Figure 4). We detect the laser spots in the 3D range sensor measurement and store the 3D coordinate of the laser spot. Note that the target for the laser ray does not need to be a flat plate, but in our setup, it was convenient because it is easy to move and the use of a plate avoids occlusion between the laser spot and the camera.

**Figure 5:**
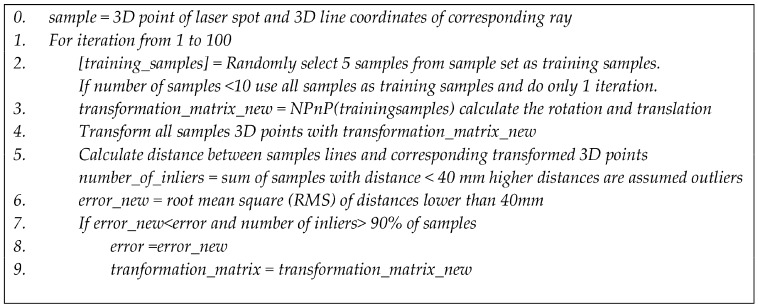
Pseudocode implementation Ransac algorithm. NPnP = Non-Perspective-n-Point.

Step 2: After Step 1, the 3D location of the laser spot in the range sensor coordinate system and the corresponding line coordinates in the laser galvanometer coordinate system are known for the N selected points. As explained in Section 2.3, the calibration comes down to solving a general NPnP problem to calculate the transformation between the galvanometric coordinate system and the range sensor coordinate system. Because 3D range sensors like Time-of-Flight cameras suffer from noise and outliers, a random consensus algorithm such as Ransac can be used to improve robustness to outliers [24,25,26,27]. This will improve the results because the NPnP algorithm uses a singular value decomposition (which is sensitive to outliers). An overview of the algorithm in pseudocode is given in Figure 5.

### 2.5. Validation Procedure

To check the consistency of proposed methodology, the stability of the algorithm is calculated with a varying number of samples (N). In the first experiment, the number of sample points is varied, while points are sampled in the complete 3D calibration space (see Section 4.1). The complete calibration/workspace is sampled by moving the plate (see Figure 4) forward and backward as is explained in Section 3 (Experimental setup). In a second experiment (see Section 4.2) the number of sample points used is fixed, but the calibration space is sampled. Sampling the calibration space is done by altering the number of board-positions used to obtain sample points.

Also, the condition number of the Hessian of the cost-function (Equation (4)) is used to evaluate how well the problem is conditioned to analyze the calibration. The Hessian can be easily calculated by using algorithmic differentiation. In this work, the toolbox described of [28] is used to calculate the Hessian. 

(4) $$H=\frac{\delta^{2}E}{\delta \alpha \delta \alpha }$$

## 3. Experimental Setup

In this section, two experimental setups are described. The first setup is used to calibrate the system with our methodology and to test the stability of the methodology. A second setup uses a calibration (obtained from the first setup) to aim the laser beam at predefined target points on an object. The accuracy of the calibration is tested with this setup.

### 3.1. Calibration Setup (Validation Setup)

**Figure 6:**
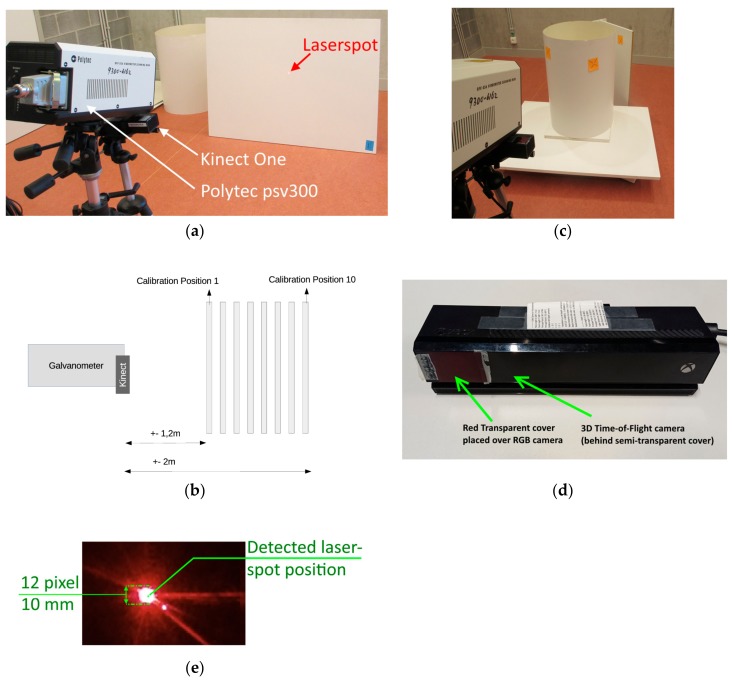
(**a**) Experimental setup for calibrating the system (Polytec PSV 300, Kinect for Xbox One). (**b**) The calibration plate is moved to different positions. (**c**) Experimental setup for comparing the 3D calibration with a standard two-dimensional (2D) calibration. (**d**) Kinect for Xbox One, the RGB camera is covered by a red semitransparent plastic for easier laser spot detection. (**e**) Result of laser spot detection.

For acquiring 3D samples of laser spots, a white plate is used (Figure 6a). Note that other calibration objects can be used, as long as the samples can be detected properly by the range sensor. The plate is positioned at different distances to the laser galvanometer (Figure 6b) and the laser galvanometer is aimed towards this plate. To sample the complete 3D work/calibration space, the plate is moved 10 times. For validating the methodology, 2000 samples are taken per plate-position (the number of samples is selected such that we are able to study the statistical distribution of the errors). The number of samples is high for validation purposes, whereas a standard calibration requires fewer samples. The calibration plates are placed between 1.2 and 2 m from the LDV and Kinect One.

The following setup was used to obtain the sample points:

- Polytec laser Doppler vibrometer PSV 300 (LDV) (laser galvanometric system)
  ○ Intrinsic parameters obtained with the method of [16]
  ○ The galvanometer mirror positions are controlled in Matlab with two analog outputs of a National Instruments USB 6343 card.
- Kinect One (Kinect for Xbox One Time-of-Flight and RGB camera) [19]
  ○ Intrinsic parameters from factory calibration
  ○ Controlled in Matlab with the Kin2 toolbox [19]
- Calibration plate (standard Melamine White Panel)
  ○ Moved 10 times to get samples in the complete workspace (a standard calibration needs fewer positions)
  ○ 2000 samples per position

To detect the laser spot the integrated RGB camera of the Kinect One is used. The RGB camera is covered by a red semitransparent cover for easier laser spot detection (Figure 6d). The Kinect One shutter time and/or diaphragm are not configurable. Therefore, without the transparent cover, the laser spot overexposes parts of the image. The laser spot is detected using background subtraction in the RGB image and then converted to 3D coordinates using the Kinect One internal calibration between the 3D Time-of-Flight sensor and RGB camera. As a reference frame for the background subtraction, an image is taken with the laser spot placed outside the field of view of the camera. The reference frame is subtracted from an image with the laser spot. This image is then thresholded so that the laser spot appears as 1 and the background as 0. The center of the laser spot is calculated by taking the median of the position of foreground (1) values. A result of the detection is shown in Figure 6e.

Experiments on validating the methodology are described in Section 4.1 and Section 4.2.

### 3.2. Setup for Comparing the Aiming of 2D Calibration and 3D Calibration

A second setup is used to compare our 3D calibration methodology with the methodology originally used in the standard Polytec vibrometer software and [3]. This setup consists of a calibrated galvanometric setup (vibrometer) and Kinect One that points the laser towards a cardboard barrel (diameter 650 mm) (Figure 6c). Experiments with this setup are described in Section 4.4.

## 4. Experiments and Methodology

During experiments, a large dataset is built to do an accuracy and stability analysis of the calibration process. We use this stability analysis to derive a set of specific recommendations for the calibration process for a given accuracy. These recommendations are used to do the final experiment that compares the accuracy of the 3D calibration methodology with a 2D calibration.

### 4.1. Validating the Number of Samples

**Figure 7:**
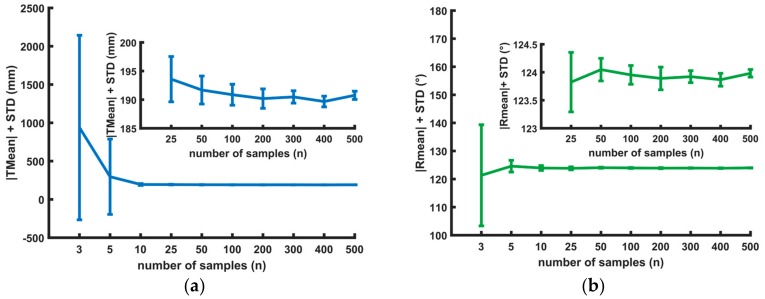
(**a**) Visualization of the amplitude of the translation (T_mean_) and (**b**) amplitude of the rotation angle (R_mean_) (axis-angle representation). Each test is repeated 50 times with n (random) sample points. The 68% confidence interval (2 times the standard deviation) is visualized with whiskers.

The calibration between the Kinect One and LDV is calculated using a subset of the samples that increases in size (3 to 500). In Figure 7, different calibration results with a different number of samples are listed. For each sample size, the calibration is repeated 50 times. The samples are randomly selected from the complete dataset containing all samples of all the positions of the calibration board.

Although in theory the calibration only needs three sample points (due to the nature of the NPnP-problem), Figure 7 shows that using only three points gives an unacceptably high standard deviation for both translation and rotation.

When 25 or more samples are used, the standard deviation of the translation is below 10 mm and the standard deviation of the rotation is below 1 degree.

In the next section, a stability analysis is executed with 100 sample points and the number of calibration board positions is altered.

### 4.2. Validating Sampling of the 3D Workspace

In this section, a stability analysis on the calibration is done by altering the number of calibration board-positions and using 100 random sample points. By altering the number of board positions, the sampling of the 3D workspace is changed. Also, the condition number of the Hessian of the energy function (Equation (1)) is calculated and shown in Table 1 together with the Root Mean Square (RMS) value of the energy function.

**Figure 8:**
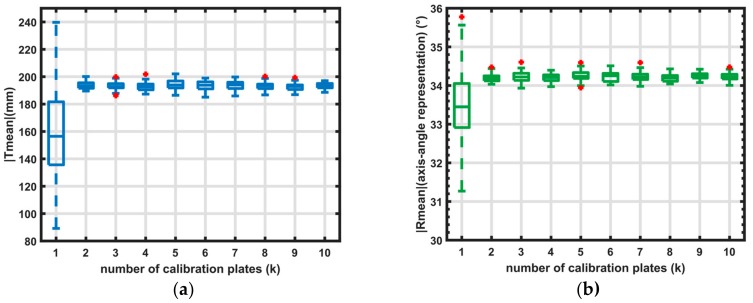
Boxplot of calibration. Each dataset is built from k calibration plates; the calibration is repeated 50 times with 100 random points. The bottom and top edges of the box indicate the 25th and 75th percentiles, respectively. The whiskers (dotted lines) extend to the most extreme data points (Translation or Rotation) that are not considered outliers. The outliers are plotted as a (red) plus symbol. (**a**) Visualisation of translation (Tmean) and (**b**) rotation angle (R_mean_) (axis-angle representation).

The graphs in Figure 8 show that sampling on one plate gives large differences of the coordinate transformation (more than 80 mm and 3 degrees). When more than one plate is used, the calibration is accurate (with differences below 20 mm and 1°).

Table 1 also gives the RMS (Root Mean Square) values of the energy function E (see Equation (1), Figure 1). The RMS value is only presented for one (random) calibration per dataset. Given only this RMS value (and standard deviation), the first calibration (Table 1—one calibration plate) would seemingly give the best results. This shows that the RMS value should be used with care (the results in Figure 8 clearly show that de-calibration using only one plate can be highly inaccurate). In this case, the calibration is over-fitted on the dataset. This is also shown in the condition number of the Hessian (see Equation (3)) where the condition number is high (>4000) when using one plate, and lower (<300) when using multiple plates.

### 4.3. Discussion and Recommendations

It is better to use samples of the whole workspace then samples on one plane. The RMS error (and standard deviation) is not a good indicator to analyze the calibration because in our experiments, the RMS error was lower when samples located on one plane were used. A better tool to analyze the performance of the calibration procedure is the condition number of the Hessian of the energy-function (Equation (1)). In our tests, unstable calibrations have a condition number of around 4000 and higher, while more stable calibrations have a condition number below 300.

### 4.4. Comparison with 2D Calibration

**Figure 9:**
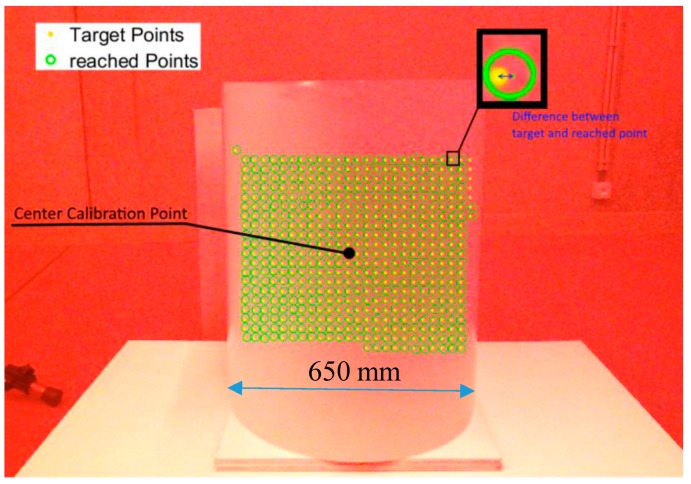
Example of the target and reached points on a cardboard barrel with the 3D method. Detected/reached points are defined by circles, target points by points. Some points were not detected.

In this section, we compare the accuracy of the proposed 3D calibration and the 2D method used in [3]. The 3D calibration uses a calibration with 100 sample points divided over 2 positions of the calibration board. The 2D method is also executed with 100 points but calibrated directly on the object (cardboard barrel, Figure 9) as is done in [5].

The two methods are compared by aiming the laser galvanometer to 500 pre-defined points. The points are defined in the RGB-camera coordinate system for the 2D method (pixel). For the 3D method, the points are defined in the 3D coordinate system of the camera.

**Figure 10:**
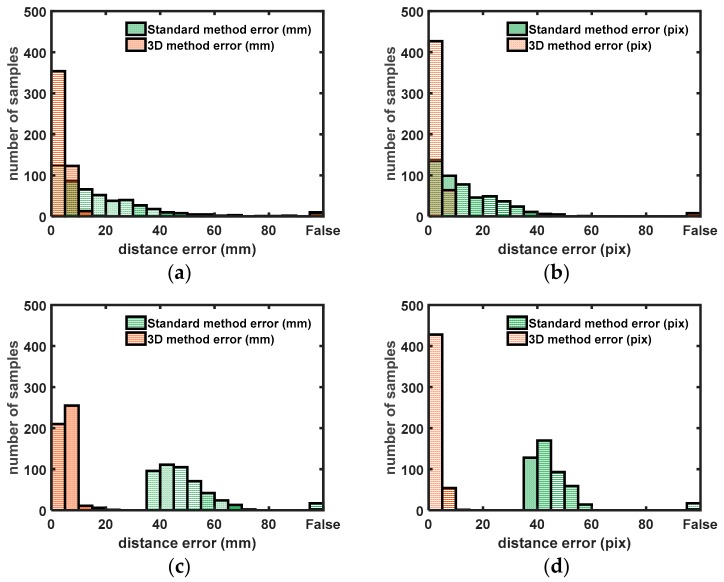
Histograms containing the difference between the measured laser spot and the input laser spot. “False” values are laser spots that are not detected (out of search range). (**a**,**b**) Distance errors (pixel, mm) when the barrel was not moved after (2D) calibration. (**c**,**d**) Distance errors (pixel, mm) when the barrel was moved 1 m away from the original position.

Figure 10 shows an example of the target points and reached (detected) points. In Figure 10, the distance between the target and reached points is shown. The difference is given in millimeter and pixel units.

The results show that 80% of the aiming errors using 3D extrinsic calibration are below 5 mm and 99% of the measurements are below 10 mm. For the 2D calibration, only 25% of the measurements have an accuracy below 5 mm.

In the second test, the barrel is moved 1 m away from the galvanometric setup. With the 3D calibration, 60% of the measurements are below 5 mm and 95% are lower than 10 mm. This is less than the first barrel position due to the lower spatial resolution of the 3D sensor when objects are located further away from the sensor. The first position gave a spatial resolution of approximately 3 mm (on the barrel), where the second position has a spatial resolution of 7 mm. The 2D method only gives errors above 35 mm, which is significantly worse than the 3D method. Note that any point with a distance error greater than 60 mm is identified as a ‘False’ point. A point can be false it detected wrongly due to distortion of the laser spot near the edges of the barrel. The point can also be identified as false when de-detection is good but the positioning error is high.

**Figure 11:**
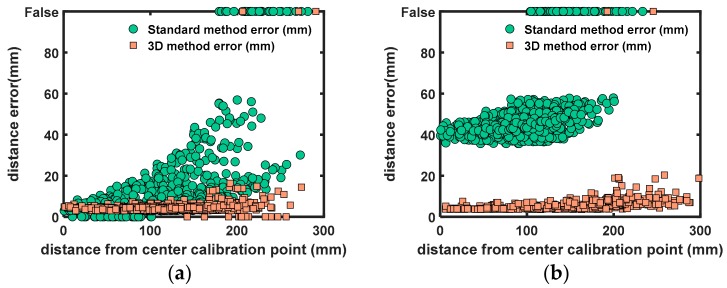
Scatter plots with differences between the measured laser spots and the input laser spots in relation with the distance to the center calibration point. “False” values are laser spots that are not detected (out of search range). (**a**) Distance errors (mm) when the barrel was not moved after 2D calibration. (**b**) Distance errors (mm) when the barrel was moved 1 m away from the original position.

Figure 11 shows the positioning error in relation to the distance of the center calibration point (of the 2D calibration, see Figure 9). The center calibration point lies approximately in the center line of the cardboard barrel. The figure shows that the positioning error with the 3D calibration only deteriorates slightly when moving further away from the center. The positioning error with the 2D method deteriorates up to 60 mm when moving further away from the center. This is expected because the 2D method assumes flat surfaces, which is not the case.

## 5. Conclusions

The solution of the NPnP problem can solve the extrinsic calibration between a laser galvanometric system and a 3D range sensor. Although the solution minimizes the RMS error of the distance between the laser rays and selected 3D point in the range sensor coordinate system, the calibration with the lowest RMS value is not necessarily the best calibration. When the workspace is not sampled properly (e.g., samples on one calibration plate), the problem is ill-posed (large condition number of the Hessian in Equation (4)) and sensitive to over-fitting. When using a calibration (with a properly sampled workspace) to aim towards selected points, the setup using a Polytec laser Doppler vibrometer and Kinect One can aim with an accuracy of 10 mm (95% of measurements). This is significantly better than that of the original 2D method which can give large distance errors (higher than 35 mm).

From our extensive stability analysis, we can derive a specific set of recommendations for obtaining a good calibration with a minimum set of samples:

- The calibration should be performed with at least 100 laser spot samples.
- The calibration should use measurements from at least two different plane positions in the workspace.

The condition number of Hessian (Equation (4)) can be used to evaluate the calibration. In our experiments when samples are taken in at least two different planes in the workspace, the condition number was always below 300 (resulting in an acceptable accuracy).

## Figures and Tables

**Table 1:** RMS error metric and standard deviation of a single calibration (100 points) with a different number of calibration plates. Also, the mean condition number (closer to 1 is better) of the Hessian of the cost function is given.

Number of Calibration Plates		1	2	3	4	5	6	7	8	9	10
	RMS (mm)	4.8	8.5	17.2	16.5	17.5	14.0	13.9	13.6	12.4	19.0
	STD (mm)	3.2	7.5	16.0	15.4	16.4	12.9	12.8	12.5	11.3	18.0
Condition number	Mean	4.2 × 10^3^	198	188	245	281	290	239	192	206	182
